# Homogenization Theory for the Prediction of Obstructed Solute Diffusivity in Macromolecular Solutions

**DOI:** 10.1371/journal.pone.0146093

**Published:** 2016-01-05

**Authors:** Preston Donovan, Yasaman Chehreghanianzabi, Muruhan Rathinam, Silviya Petrova Zustiak

**Affiliations:** 1 Department of Mathematics and Statistics, University of Maryland, Baltimore County, Baltimore, Maryland, United States of America; 2 Department of Biomedical Engineering, Saint Louis University, St. Louis, Missouri, United States of America; Queensland University of Technology, AUSTRALIA

## Abstract

The study of diffusion in macromolecular solutions is important in many biomedical applications such as separations, drug delivery, and cell encapsulation, and key for many biological processes such as protein assembly and interstitial transport. Not surprisingly, multiple models for the a-priori prediction of diffusion in macromolecular environments have been proposed. However, most models include parameters that are not readily measurable, are specific to the polymer-solute-solvent system, or are fitted and do not have a physical meaning. Here, for the first time, we develop a homogenization theory framework for the prediction of effective solute diffusivity in macromolecular environments based on physical parameters that are easily measurable and not specific to the macromolecule-solute-solvent system. Homogenization theory is useful for situations where knowledge of fine-scale parameters is used to predict bulk system behavior. As a first approximation, we focus on a model where the solute is subjected to obstructed diffusion via stationary spherical obstacles. We find that the homogenization theory results agree well with computationally more expensive Monte Carlo simulations. Moreover, the homogenization theory agrees with effective diffusivities of a solute in dilute and semi-dilute polymer solutions measured using fluorescence correlation spectroscopy. Lastly, we provide a mathematical formula for the effective diffusivity in terms of a non-dimensional and easily measurable geometric system parameter.

## Introduction

The study of diffusion of solutes such as small molecules or globular proteins in aqueous macromolecular solutions is important in many biological, biomedical, and biopharmaceutical fields. For example, hindered diffusion in macromolecular systems influences protein assembly [1], intracellular and interstitial transport [2, 3], the design of cell scaffolds for tissue engineering applications [4], and drug or protein release from drug delivery devices, [5, 6] among others. Accordingly, tracer diffusion in solutions of macromolecules has been a focus of research for several decades [7, 8]. While great strides have been made, diffusion, being a dynamic process, is difficult to measure in real time [9, 10] and challenging to accurately predict a-priori [11, 12]. Underpinning their importance in multiple fields, many models have been developed for the prediction of diffusion in macromolecular systems [11, 13–23]. However, most theoretical models include parameters that are not easily measurable, are specific for the solute-macromolecule-solvent system, or depend on fitted parameters that lack physical meaning. Thus, models that utilize readily available or measurable system parameters would be of high utility to researchers in the field.

In the context of general heterogeneous media, a vast body of literature exists for obtaining effective homogeneous macroscopic diffusivity based on fine-scale diffusivities of heterogeneous media [24, 25], which goes as far back as the work of none other than Maxwell [26]. The early work considered inclusions of spherical or other simple shapes of one type of medium embedded in another medium, and focused on the case when the volume fraction of the inclusions was very small. A relatively recent theory, known as homogenization theory, generalizes this earlier body of work [27–33] and is backed by rigorous mathematical limits. The inclusions considered in this theory can be far more general in shape and the volume fraction of inclusions need not be small.

In this manuscript, we develop a framework based on homogenization theory for the prediction of solute diffusivity in macromolecular environments and validate the theory with experimental data obtained by fluorescence correlation spectroscopy (FCS).

The homogenization theory provides a simple large scale homogeneous approximation of a medium which is heterogeneous in the fine-scale, with respect to a property that is governed by a mathematical equation. To the best of our knowledge, the applicability of this powerful theory towards the prediction of solute transport in macromolecular environments has not been explored previously with the backing of experimental measurements. The homogenization theory usually applies to two forms of microscopic media: periodic or random. The periodic version of the theory applies to heterogeneous but periodic spatial structures where the periodic length is very small compared to the spatial extent of interest. The random version of the theory applies to heterogeneous media with fine and random structure that possess certain characteristics (stationarity and ergodicity) and the characteristic length of the fine structure is very small compared to the spatial extent of interest. The homogenization theory provides a rigorous mathematical limit of the property under study as the scale separation ratio *ϵ* (the ratio of the periodic or characteristic length of the fine spatial structure to the length of the spatial region of interest) approaches 0. The validity of the resulting approximation depends on whether the scale separation is significant (*ϵ* sufficiently small). An important message that is learned from the homogenization theory is that the effective large scale property is often not a straightforward spatial average of the fine-scale property of the heterogeneous medium [30, 33]. In fact the effective large scale property is obtained via solving a partial differential equation inside a periodic cell—the so-called “unit cell problem”- and then computing an average of certain derivatives of the solution. This observation is one of the key reasons for our use of the homogenization approach as it provides a rigorous result once an appropriate periodic microscopic model is decided upon.

Here we use the periodic homogenization theory due to its simplicity. While the homogenization theory provides an overarching theme, the choice of the periodic microscopic model is important. As we are studying the diffusion of a solute in a macromolecular solution, as a first approximation, we investigate a model consisting of periodically placed stationary and spherical macromolecules acting as impenetrable obstacles. We also assume that there is no significant interaction between the solute and macromolecules, where the macromolecules hinder diffusion by obstruction only. This results in a model that only utilizes parameters that are readily measurable or available in the literature, thus, simplifying comparison with experimental data. For this geometry we introduce a dimensionless number, *ρ*, so that the ratio *D*_*e*_/*D*_0_ of the effective diffusivity *D*_*e*_ to the “free” diffusivity *D*_0_ in a solvent (water) without polymers, is a function of *ρ* alone. Thus, all key system parameters enter via *ρ*.

We find that the effective diffusivities predicted by the homogenization theory agree well with computationally more expensive Monte Carlo simulation results. More importantly, the predictions by the homogenization theory agree with effective diffusivities measured experimentally in dilute and semi-dilute polymer solutions. We also provide an approximating formula for *D*_*e*_/*D*_0_ predicted by the homogenization theory (see Eq (23)) as a function of *ρ*. FCS, a biophysical technique that enables in-situ, real time measurements of dynamic properties, is used to acquire the experimental data. FCS is a single molecule spectroscopic technique that measures the fluctuations of fluorescent probes in a defined confocal volume and correlates them in time to give information on diffusion times, concentrations, and interactions [34–36]. We probe various polymer-solute pairs in order to interrogate the model’s assumptions and validity. We determine that the model can provide a reasonable prediction for solute diffusivity in polymer solutions in the dilute and semi-dilute regimes; however, due to the rigid sphere assumption, the model predicts slower than experimental diffusivity in the concentrated regime. Interestingly, regardless of the spherical obstacle assumption, we observe a good agreement between model and experiment for both spherical and random coil polymer molecules. We further provide a mathematical description of the theoretical data to enable easy comparison with other models or experimental data. With the developed homogenization theory framework, we have set the stage for future theory extensions to include interaction between the solute and obstacle or other obstacle geometry that would more closely emulate the random coil structure of some macromolecules. The ability to predict diffusion a-priori based on easily measurable system parameters would enable the rational design of macromolecular systems for targeted applications, such as drug delivery, and will aid in our understanding of various transport-dependent processes.

## Theory

### Modeling Assumptions

We are concerned with the motion of a solute molecule *A* in solvent *B* where there are also polymer molecules *C* dissolved in solvent *B*. We assume that the solute A is present in low concentrations so that we expect a given solute molecule to only encounter molecules *B* and *C*. We also assume that the polymer molecules *C* are stationary and impenetrable spheres.

In the absence of polymer molecules *C*, a molecule *A* undergoes a succession of seemingly random changes in its direction of motion as well as its velocity due to successive interactions or collisions with the numerous surrounding molecules *B* of the solvent, where the interactions themselves tend to occur after seemingly random durations of time. We may regard the motion of the solute in between successive changes in direction and velocity as steps in a random walk. Under certain assumptions on the steps of this random walk over a time scale (or equivalently a length scale) that is sufficiently large, the solute molecule *A* would have undergone several steps of the random walk and the central limit theorem may be evoked to approximate the random walk by the mathematically idealized Brownian motion also known as the Wiener process [37, 38]. Then the probability density *p*(*x*, *t*) of finding a given molecule A at position *x* at time *t* is governed by the the partial differential equation (PDE) of diffusion

$$\begin{array}{c} \frac{\partial }{\partial t}p\left(x,t\right)=D_{0}\Delta p\left(x,t\right), \end{array}$$ (1)

where Δ is the Laplacian and the scalar *D*_0_ is the diffusivity, which depends on the solute *A* and the solvent *B* among other things. If the motion is happening in a confined region Ω such as a container then the PDE Eq (1) is solved on the spatial domain Ω with the no-flux boundary conditions on the boundary ∂Ω of Ω. The no-flux conditions are expressed by

$$\begin{array}{c} \nabla p(x,t)\cdot n(x)=0 \end{array}$$ (2)

where ∇*p* is the spatial gradient of *p* and *n*(*x*) is the vector normal to the boundary ∂Ω of Ω at a location *x* on the boundary.

In the presence of large polymer molecules *C* in solution, a given solute molecule *A* will undergo collisions with both the solvent molecules *B* as well as the polymer molecules *C*. On average, if the solute molecule *A* undergoes several collisions with the solvent molecules *B* in between collisions with polymer molecules *C*, we may assume the motion of the solute molecule *A* to be a Wiener process with specular reflections at the molecules *C*. Under our assumption that the polymer molecules are stationary and impenetrable spheres of radius *R*, we have a reflected Wiener process describe the motion of the center of molecule *A* where the reflections happen at the surface of the stationary spherical obstructions of radius *R*+*a*. In this case the probability density *p*(*x*, *t*) of finding a given solute molecule *A* is still given by the same PDE Eq (1). However, the domain of the PDE is the domain Ω′, which excludes from Ω the spherical obstructed regions. The boundary conditions Eq (2), in addition to the external boundary ∂Ω, shall also apply on the internal boundaries, which are the surfaces of the spherical obstructions.

If the spatial length scale *L*′ of Ω is sufficiently large compared to the characteristic spacing *L* between the spherical obstructions, one may expect to obtain some approximations for the diffusive behavior of solute *A*. In order to apply the periodic version of the homogenization theory we assume that the polymer molecules are spaced periodically (simple cubic arrangement) with center-to-center distance *L*. The homogenization theory of periodic structures predicts that when *L* is much smaller than *L*′ we may treat the resulting motion of solute *A* as (normal) diffusion in a homogeneous medium (without obstructions) which has an effective diffusivity *D*_*e*_ that differs from *D*_0_. A more realistic assumption on the placement of the spherical obstructions is that they are randomly placed (subject to non-overlap) with uniform probability density. This leads to the homogenization theory for random media. We expect this approach to yield a similar value for *D*_*e*_.

### Parameter *ρ*

**Fig 1:**
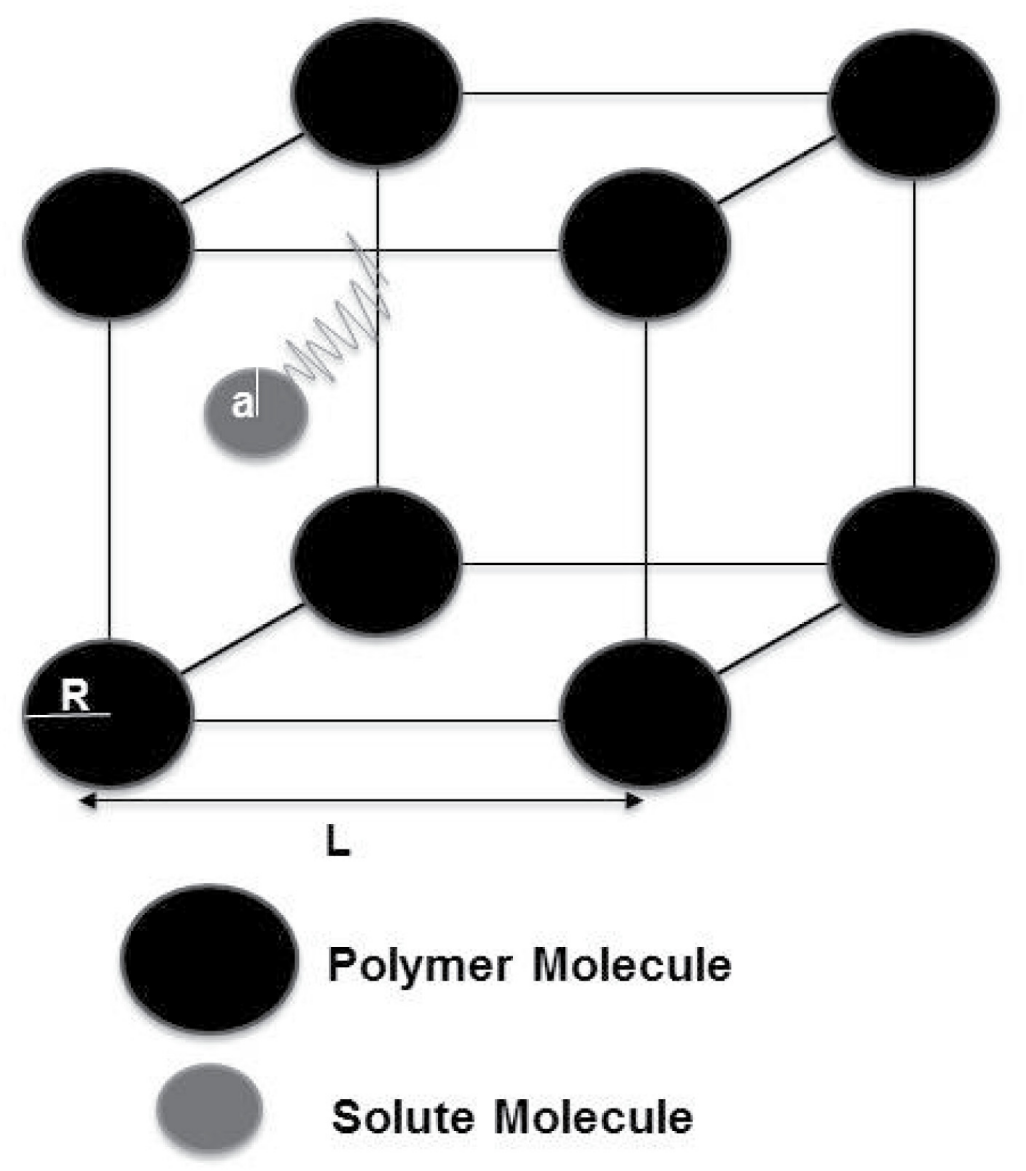
Microscopic model.

We define *ρ*, a dimensionless number, as

$$\begin{array}{c} \rho =\frac{2(R+a)}{L}, \end{array}$$ (3)

where *a* is the hydrodynamic radius of the solute, *R* is the hydrodynamic radius of the polymer molecule and *L* is the average center-to-center distance between polymer molecules in solution (see Fig 1). The values for *R* and *a* were obtained from the literature and are summarized in Table 1.

**Table 1:** Summary of polymer and solute properties.

**Polymer**	**M**_**W**_ **(kDa)**	**Hydrodynamic radius**, **R** **(nm)**	c^*^ **(%)**
Dextran 500	500	15.9 [39]	2.5
Dextran 70	70	5.8 [40]	6.9
Ficoll	400	8.0 [41]	9.1
**Solute**	**M**_**W**_ **(kDa)**	**Hydrodynamic radius**, **R** **(nm)**	
RNase	13.7	1.8 [42]	-

The center-to-center distance *L* between two polymer molecules is calculated from geometric considerations assuming a simple cubic arrangement by

$$\begin{array}{c} L={\left(\frac{M_{W}}{c\times N_{A}}\right)}^{\frac{1}{3}}, \end{array}$$ (4)

where *M*_*W*_ is the molecular weight of the polymer, *c* is the concentration of the polymer solution (in weight per volume) and *N*_*A*_ is the Avogadro number (6.022 × 10^23^ particles/mol). An example of the calculated *L* and *ρ* values for a certain polymer-solute system is given in Table 2.

**Table 2:** Summary of calculated center-to-center distance, *L*, and *ρ* values for a representative polymer-solute pair, namely Dextran500 and fluorescently labelled RNase.

Dextran500 conc. (mg/ml)	*L*(nm)	*ρ*
0.2	160.7	0.219
0.4	127.54	0.276
0.6	111.41	0.316
0.8	101.23	0.348
1	93.97	0.375
3	65.15	0.541
5	54.95	0.642
7	49.12	0.718
10	43.62	0.809
18	35.82	0.985
25	32.14	1.098
50	25.5	1.384

### Homogenization Theory

**Fig 2:**
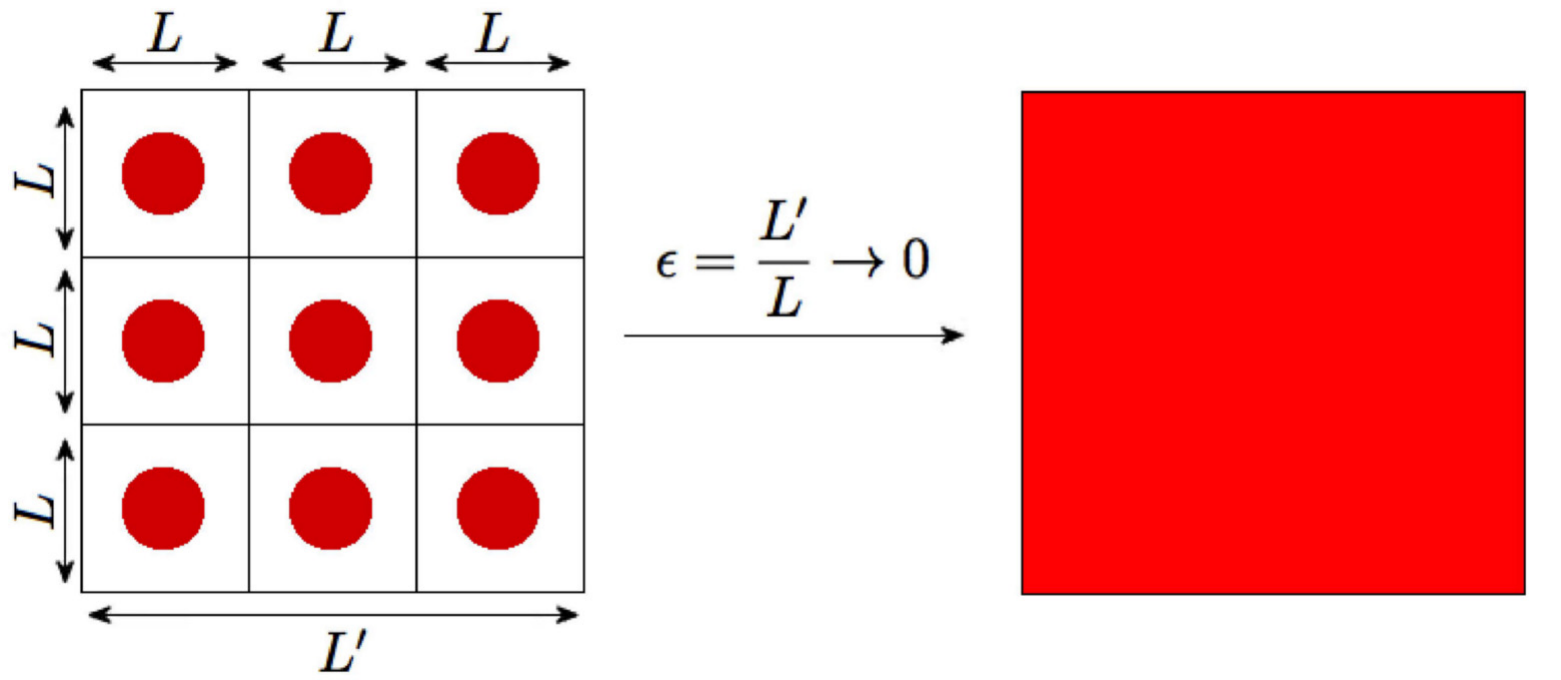
Visualization of homogenization theory limit.

Here we summarize the periodic homogenization theory as applied to our problem [43]. Let Ω denote the domain of interest in which we are observing the diffusion phenomenon and suppose *L*′ is the length characterizing Ω. We introduce a scaling factor, *ϵ* = *L*/*L*′, where *L* is the spacing between the periodic obstructions. Let Ω_*ϵ*_ be the region with the periodic spherical obstructions of radius *R*+*a* removed from Ω, as in Fig 2. We note that spheres of radius *R*+*a* represent the obstructed regions that the center of the solute molecule may not enter. The probability density *p*_*ϵ*_(*x*, *t*) of finding the solute molecule satisfies

$$\begin{array}{ccc} \frac{\partial }{\partial t}p_{\epsilon }\left(x,t\right)-D_{0}\Delta p_{\epsilon }\left(x,t\right) & = & 0\;\text{on}\;\Omega_{\epsilon },\\ \nabla p_{\epsilon }\left(x,t\right)\cdot n\left(x\right) & = & 0\;\text{on}\;\Gamma_{\epsilon },\\ \nabla p_{\epsilon }\left(x,t\right)\cdot n\left(x\right) & = & 0\;\text{on}\;\partial \Omega , \end{array}$$ (5)

where *Γ*_*ϵ*_ is the boundary between Ω_*ϵ*_ and the spherical obstructions and ∂Ω is the boundary of Ω. We note that since the domain in which Eq (5) is solved varies with *ϵ*, so does the solution. According to the homogenization theory, *p*_*ϵ*_ converges to *p*_0_, as *ϵ* → 0, where the limit *p*_0_ satisfies the PDE

$$\begin{array}{ccc} \frac{\partial }{\partial t}p_{0}\left(x,t\right)-D_{e}\Delta p_{0}\left(x,t\right) & = & 0\;\text{on}\;\Omega ,\\ \nabla p_{0}\left(x,t\right)\cdot n\left(x\right) & = & 0\;\text{on}\;\partial \Omega , \end{array}$$ (6)

where the effective diffusivity coefficient *D*_*e*_ is obtained from the solution to the so-called unit-cell problem.

Define $\bar{\Omega }$ to be the region obtained by removing a concentric sphere of radius *ρ*/2 = (*a*+*R*)/*L* from a cubic cell of unit side length. Let *Γ* be the surface of this sphere. Define *ω*_*j*_ for *j* = 1,2,3 to be the solution of the PDE with boundary conditions:

$$\begin{array}{ccc} -\Delta \omega_{j}\left(x\right) & = & 0\;\text{on}\;\bar{\Omega },\\ \nabla \omega_{j}\left(x\right)\cdot n\left(x\right) & = & -e_{j}\cdot n\left(x\right)\;\text{on}\;\Gamma ,\\ & & \omega_{j}\left(x\right)\;\text{is}\;\text{periodic}\;\text{at}\;\text{the}\;\text{external}\;\text{boundaries}\;\text{of}\;\text{the}\;\text{cell} \end{array}$$ (7)

where *e*_*j*_ is the unit vector in the *x*_*j*_-axis direction and *n*(*x*) is the unit outward normal vector. Once *ω*_*j*_ are solved for, *D*_*e*_ is obtained by

$$\begin{array}{c} D_{e}=\frac{D_{0}}{|\bar{\Omega }|}\int_{\bar{\Omega }}[1+\frac{\partial \omega_{j}\left(x\right)}{\partial x_{j}}]dx, \end{array}$$ (8)

where *j* is 1, 2 or 3 and $|\bar{\Omega }|$ denotes the volume of the region $\bar{\Omega }$. We note that due to the symmetry, one obtains the same value for *D*_*e*_ regardless of *j* and thus only *ω*_1_ needs to be computed [33]. The solution of Eq (7) as well as the integration Eq (8) need to be performed via a suitable numerical method.

We note that this theory holds for general shapes of periodically placed obstructed regions as long as the unobstructed region is connected. However, for general shapes, the symmetry alluded to does not hold and the effective diffusivity *D*_*e*_ will be described by a matrix *D*_*e*__*ij*_ where

$$\begin{array}{c} {D_{e}}_{ij}=\frac{D_{0}}{|\bar{\Omega }|}\int_{\bar{\Omega }}[1+\frac{\partial \omega_{i}\left(x\right)}{\partial x_{j}}]dx,\;\;i,j=1,2,3. \end{array}$$ (9)

**Fig 3:**
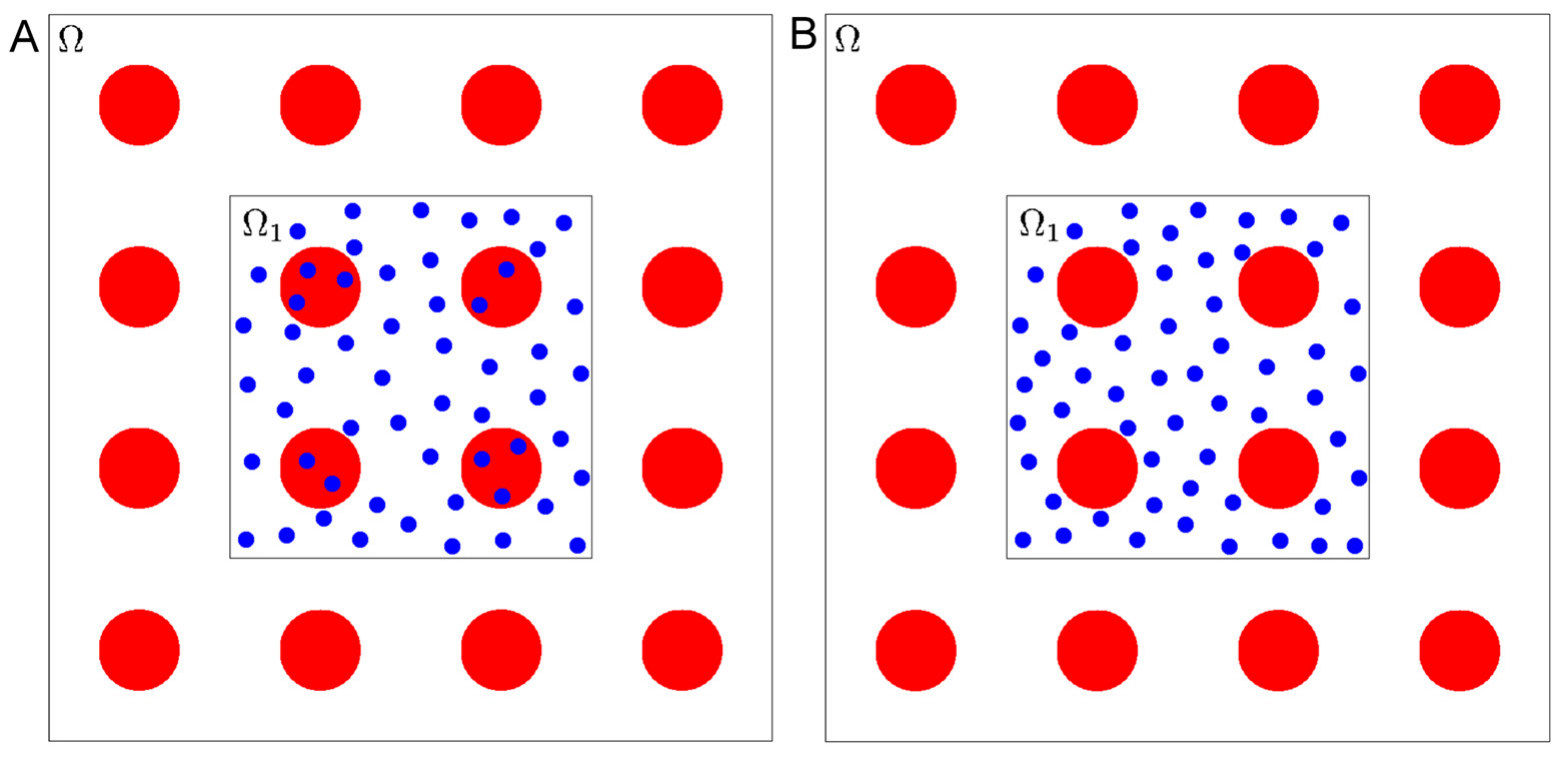
The two different initial conditions that illustrate the role of the factor 1 − *ϕ* in effective diffusivity. (a) solute evenly distributed inside Ω_1_ including the obstructed regions and (b) solute evenly distributed inside Ω_1_ excluding the obstructed regions.

It must also be noted that *D*_*e*_(1 − *ϕ*) is more commonly referred to as the effective diffusivity [28], where *ϕ* is the obstructed volume fraction. In our case, *ϕ* = *πρ*^3^/6 for *ρ* ≤ 1. Whether *D*_*e*_ (as defined by Eq (8)) or *D*_*e*_(1 − *ϕ*) is the correct effective diffusivity depends on certain details of the problem. To explain the different situations, consider the following two scenarios. Let Ω be the overall region of interest as before, with periodically placed obstructed regions with spacing *L*. Let Ω_1_ ⊂ Ω be an open connected subset, such that the length scale of Ω_1_ is comparable to that of Ω, and, thus, it is also much larger than *L*. Consider the two different initial conditions:

1. at time *t* = 0 the solute is evenly distributed inside Ω_1_, including the obstructed regions inside Ω_1_, and solute is absent outside of Ω_1_,
2. at time *t* = 0 the solute is evenly distributed inside Ω_1_, but excluding the obstructed regions inside Ω_1_, and solute is absent outside of Ω_1_,

and further assume that the total quantity of solute inside Ω_1_ at *t* = 0 is the same in both situations (see Fig 3). Given these two different initial conditions, we want to know how the solute diffuses out into rest of Ω for *t* ≥ 0. If one were to approximate the entire region Ω_1_ with an effective diffusivity, one must use *D*_*e*_ as defined by Eq (8) in the second scenario while one must use *D*_*e*_(1 − *ϕ*) in the first scenario. The intuition is clear when one considers the stochastic motion of the solutes: in the second scenario all solutes are mobile while in the first only a fraction, 1 − *ϕ*, are mobile. The reason for regarding *D*_*e*_(1 − *ϕ*) as the effective diffusivity is the following. Often in practice, the “obstructed region” may not be truly obstructed, but have very small diffusivity *δ* > 0. In this case, if the system is in steady state, the solutes will be evenly distributed inside and outside the “obstructions”. However, the time it takes to reach steady state increases without bound as *δ* approaches zero.

In terms of comparison with the FCS experiments measuring effective diffusivity in this paper, the question is whether one expects to see the solute to start out uniformly anywhere in the FCS confocal volume, or inside the confocal volume but outside of polymeric obstructions. When the solution is prepared, one expects the solute to be only present in water and not inside the polymer molecular regions, which we have assumed to be spheres. If indeed the polymers are acting as regions of extremely small non-zero diffusivity *δ* > 0, then one can expect the steady state to be reached a very long time after the solution is prepared. In this case, since there are only a few solute molecules present in the entire FCS confocal volume, one would expect a two component fit showing low diffusivity for some solute molecules and larger diffusivity for the other. The fact that a two component fit was not observed suggests that, in the practical time frame of the FCS experiments, the solutes did not have time to effectively enter the polymeric regions. Thus, we expect the correct effective diffusivity to use is *D*_*e*_ defined by Eq (8).

### Monte Carlo Simulations and Effective Diffusivity

The reflected Wiener process description of the motion of a solute molecule *A* may be expected to be reasonable if on average the solute undergoes several collisions with solvent molecules *B* in between collisions with polymer molecules *C*. In order for this to be valid, one expects the mean path length of the solute *A* in solvent *B* should considerably smaller than the length of the gap between the spheres of obstruction with radius *R*+*a*.

To verify the validity of the reflected Wiener process model, we compare Monte Carlo simulations of the reflected Wiener process with Monte Carlo simulations of a random walk model, which we call the kinetic model. In this model we assume that (the center of) the solute molecule *A* follows a random walk consisting of rectilinear motions with exponentially distributed lengths, exponentially distributed time durations and uniformly distributed directions. More details are provided in the Computational Methods section.

Both the Wiener process and the kinetic model simulations can be used to test whether the motion of the solute molecule over long length scales behaves like unobstructed normal diffusion with an effective diffusivity *D*_*e*_ that is different from the unobstructed diffusivity *D*. If the solute motion follows a normal diffusion, the mean square displacement must be linear in time with a slope *D*_*e*_/6.

Recall that the predictions of the homogenization theory are only valid in the limit when *L*′ is significantly larger than *L*. Thus we expect agreement of Homogenization theory results with the reflected Wiener process simulations when *L*′ is sufficiently larger than *L*. As described in the section on the comparison of Monte Carlo and homogenization computations, agreement was seen even when *L*′ was only a few multiples of *L*.

### Anomalous versus normal diffusion

The term normal diffusion applies to the Wiener process. One property of a Wiener process *X*(*t*) is that the mean square displacement (MSD) given by *MSD*(*t*) = *E*(‖*X*(*t*) − *X*(0)‖^2^) (where *E* is the expected value) is a linear function of time *t*:

$$\begin{array}{c} MSD(t)=Ct, \end{array}$$ (10)

where *C* is a constant. For the Wiener process (or normal diffusion) in *d* dimensions, *C* = 2*dD*, where *D* is the diffusivity. Generalizing the above equation by replacing *t* with a different power *α* other than 1 leads to

$$\begin{array}{c} MSD\left(t\right)=Ct^{\alpha }, \end{array}$$ (11)

and processes *X*(*t*) that satisfy this criterion with *α* ≠ 1 are referred to as anomalous diffusion [44, 45]. When *α* > 1, the behavior is termed superdiffusive and for *α* < 1 it is termed subdiffusive. However, it must be noted that the MSD curve does not uniquely characterize a stochastic process unless further assumptions are made about the time evolution equation for the probability density function. See [22, 46] for instance, where a model based on fractional differential equations is described. In general, a stochastic process may not satisfy Eq (11) for all *t*, but large *t* or small *t* behavior may be still be given Eq (11). In other words, if

$$\begin{array}{c} \lim_{t\to \infty }MSD\left(t\right)/t^{\alpha }=C, \end{array}$$ (12)

or

$$\begin{array}{c} \lim_{t\to 0+}MSD\left(t\right)/t^{\alpha }=C, \end{array}$$ (13)

where *C* > 0, then the process is said to be anomalous diffusion for large or respectively small time scales. Instead of considering rigorous *t* → 0+ or *t* → ∞ limits, one may consider different approximations over different time intervals of the form:

$$\begin{array}{c} MSD\left(t\right)=C_{i}t_{i}^{\alpha },\;t_{i}\leq t\leq t_{i+1}. \end{array}$$ (14)

The plot of log(*MSD*(*t*)/*t*) against log(*t*) is used in [47] as a simple visual aid to differentiate normal versus anomalous diffusion. If the form of relationship in Eq (14) holds, then one gets

$$\begin{array}{c} \log\left(MSD\left(t\right)/t\right)=\left(\alpha_{i}-1\right)\log\left(t\right)+\log\left(C_{i}\right),\;t_{i}\leq t\leq t_{i+1}. \end{array}$$ (15)

If the plot of log(*MSD*(*t*)/*t*) against log(*t*) is obtained via Monte Carlo simulations, one may apply continuous and piecewise linear fit to identify different temporal regions of different diffusive behaviors. See for instance [46–49] for Monte Carlo studies of random walk models on obstructed lattices.

In the case of the reflected Wiener process considered by us, one theoretically expects both small *t* and large *t* limits to show normal diffusion. In other words, one expects

$$\begin{array}{c} \lim_{t\to 0+}MSD\left(t\right)/t=6D_{0},\;\;\;\lim_{t\to \infty }MSD\left(t\right)/t=6D_{e}. \end{array}$$ (16)

Intuitively, for small time *t*, the process does not see the obstructions and hence behaves like the (non-reflected) regular Wiener process with free diffusivity *D*_0_. On the other hand, homogenization theory predicts that over large length scales or equivalently time scales, the process behaves like a regular Wiener process with a smaller diffusivity *D*_*e*_ as given by Eq (8). As *MSD*(*t*) is continuous and smooth in *t*, by mathematical necessity, this curve will be non-linear (curved) in order to satisfy the large *t* and small *t* limits. This is indeed observed in our Monte Carlo simulations as we shall see later.

## Materials and Methods

### Computational Methods

The entirety of the computational results were collected on a Macbook Pro with a 2.6 GHz Intel Core i7 processor and 8 GB of RAM. The Monte Carlo simulations were programmed in C while the homogenization computations were performed in COMSOL Multiphysics, a finite element solver.

#### Monte Carlo Simulation

The C programs simulated the random walk of a solute molecule according to both the kinetic model as well as the Weiner process model. The random walk occurs in a 3-dimensional space containing periodic, stationary spheres (polymer molecules) as obstacles. We chose a time scale such that free diffusion (diffusion in the absence of obstructions) was *D*_0_ = 1 while the length scale was meant to represent nm. Across all simulations, *L* = 5 while *R* and *a* varied so that *ρ* was in the range $\left[0,\sqrt{2}\right]$. For each (*a*, *R*) pair, 100,000 simulations were completed and 250 locations were recorded at equidistant times ${\left\{t_{j}\right\}}_{j=1}^{250}$. The output of a simulation for a given (*a*, *R*) pair was a set of vectors, *X*_*i*_(*t*_*j*_), which denotes the solute molecule’s position at time *t*_*j*_ in the *i*th simulation. The mean square displacements at times *t*_*j*_ were estimated by

$$\begin{array}{c} \hat{MSD}\left(t_{j}\right)=\sum_{i=1}^{N}{∥X_{i}\left(t_{j}\right)∥}^{2}/N, \end{array}$$ (17)

where ‖*X*_*i*_(*t*_*j*_)‖ is the magnitude of the displacement and *N* = 100,000 is the number of simulations.

In the kinetic model simulation, the path length and time duration at each step were i.i.d. exponential random variables with mean *λ* and *τ*, respectively. The mean path length, *λ* = 0.20, was chosen to represent the mean free path of 0.2 nm of a water molecule in water. The value chosen for *L* is a lower bound since for typical polymer concentrations the spacing *L* is 25 to 160 nm as seen from Table 2. The value chosen for *λ* was an upper bound as the mean free path of a solute molecule (larger than a water molecule) in water would be smaller than 0.2 nm. Thus the values of *λ* and *L* were conservative in that these underestimated the mean number of random walk steps of the solute in between collisions with the polymer molecules, making it harder for the Wiener process approximation of the kinetic model to hold. We would like to note that halving the mean path length to *λ* = 0.10 yielded approximately the same results. Noting the relationship that the mean squared displacement as a function of time *t* (for the unobstructed kinetic model) is given by *λ*^2^ *t*/*τ* (for large *t*) where the *τ* is the mean time step, we chose $\tau =\frac{\lambda^{2}}{3D_{0}}$ with *D*_0_ = 1.

The Euler method was used to approximate the Wiener process with reflections and as a check we note that halving the time step of the Euler method had roughly no effect on the simulation results, verifying the accuracy of the Euler approximation. Algorithmic descriptions of the Monte Carlo simulations are provided in the Supporting Information section.

#### Homogenization Computation

COMSOL Multiphysics was used to compute solutions of Eq (7). The domain was a cube of unit side length with a spherical region concentric with the cube removed. The radius was varied over {0,0.02,0.04, …, 0.70}; this is equivalent to letting *ρ* = 0,0.04, …, 1.40. COMSOL Multiphysics was also used to compute *D*_*e*_ via Eq (8).

### Experimental Methods

#### Materials

All chemicals were used as received unless otherwise noted. Dextran500 (dextran from Leuconostoc ssp., 500 kDa), Ribonuclease A (RNase) and Atto488 NHS ester were purchased from Sigma (St Louis, MO). Dextran70 (70 kDa), Dextran270 (270 kDa) and Rhodamine 6G (R6G) were purchased from Acros Organics (New Jersey).

#### FCS measurements and data analysis

Polymer solutions of desired final concentration were prepared by dissolution of polymer in phosphate buffered saline (10 mM PBS, pH 7.4). Only polymer solutions in the dilute and semi-dilute regimes were used, i.e. below or equal to the polymer overlap concentration, c*. The overlap concentration was calculated as follows:

$$\begin{array}{c} c^{*}=\frac{M_{W}}{\frac{4}{3}\pi R_{g}^{3}N_{A}} \end{array}$$ (18)

where *R*_*g*_ is the radius of gyration of the polymer. Based on our model, we also define a geometric overlap concentration, *c*^*θ*^, as follows:

$$\begin{array}{c} c^{\theta }=\frac{M_{W}}{8R_{h}^{3}N_{A}} \end{array}$$ (19)

where *R*_*h*_ is the hydrodynamic radius of the polymer. Hence, by definition, at the geometric overlap concentration, the polymer particles would be closely packed and touching each other. To prepare polymer-solute solutions, the fluorescently labelled solute RNase (10 nM) was mixed thoroughly with the desired polymer solution or PBS only and 50 μl samples were transferred to a CoverWell perfusion chamber (Molecular Probes, Carlsbad, CA) for FCS measurements.

Measurements of solutes in PBS (to obtain *τ*_0_) and in polymer solutions (to obtain *τ*_*d*_) were performed on an FCS unit equipped with a 488–514 nm laser and a Zeiss LSM510 confocal microscope. All measurements were performed at 22°C. Acquisition times of 200 s were used to optimize signal-to-noise ratio. Low laser intensity was used throughout to avoid activation of the fluorophore triplet states as well as photo bleaching. The instrument was calibrated with R6G (diffusivity for R6G = 2.8 × 10^−10^ m^2^ s^−1^ in water). The radius of the laser beam spot was estimated to be 208 nm. The resultant autocorrelation functions were fitted with the model depicted in Eq (20) which describes normal diffusion of a single monodisperse fluorophore [34]:

$$\begin{array}{c} G\left(\tau \right)=1+\frac{1}{N}\frac{1}{\left(1+\frac{\tau }{\tau_{d}}\right)}\frac{1}{{\left(1+p\frac{\tau }{\tau_{d}}\right)}^{0.5}} \end{array}$$ (20)

where *N* is the average particle number in the detection volume, *τ* is the delay time, *τ*_*d*_ is the characteristic diffusion time, and *p* = (*r*_0_/*z*_0_)^2^ is an instrumental constant (where *r*_0_ and *z*_0_ are the radius and axial length of the focused laser beam spot, respectively). Assuming a three-dimensional Gaussian profile of the excitation beam, *τ*_*d*_ can be related to diffusivity, *D*, by the following equation:

$$\begin{array}{c} \tau_{d}=\frac{r_{0}^{2}}{4D} \end{array}$$ (21)

#### RNase labeling

RNase was labeled with Atto488 NHS ester with 52% labeling efficiency following the manufacturer’s procedures. Briefly, 10 mg/ml RNase in PBS was reacted with Atto488 NHS ester at 1:2 molar ratio of RNase to Atto488 NHS ester for 2 h at room temperature. Unbound fluorophore was removed with Dye Removal Columns with >95% efficiency. The final conjugate was used immediately or lyophilized and stored at -20°C for long term storage.

#### Statistics

All experimental data is reported as the average ±SD from three to six independent experiments with minimum of four samples per experiment. Coefficient of determination, *R*^2^, was used to compare the goodness of the fit between the experimental data and the model:

$$\begin{array}{c} R^{2}=1-\frac{SS_{res}}{SS_{tot}} \end{array}$$ (22)

where *SS*_*res*_ is the residual sum of squares and *SS*_*tot*_ is the total sum of squares.

## Results and Discussion

### Comparison of Monte Carlo and homogenization computations

In our Monte Carlo simulations the length scale was chosen to be nm while the time scale was chosen so that the free diffusion coefficient was 1. The lengths *a*, *R* and *L* correspond to solute radius, radius of polymer molecule in solution when interpreted as a rigid sphere and the center-to-center spacing of the polymer molecules in solution, respectively. As the solute and polymer molecules are not spheres in reality, we used their hydrodynamic radii (*R* = *R*_*h*_). These quantities are known for any given polymer-solute pair and given polymer concentration and hence we can interpret our simulation results in terms of these lengths.

**Fig 4:**
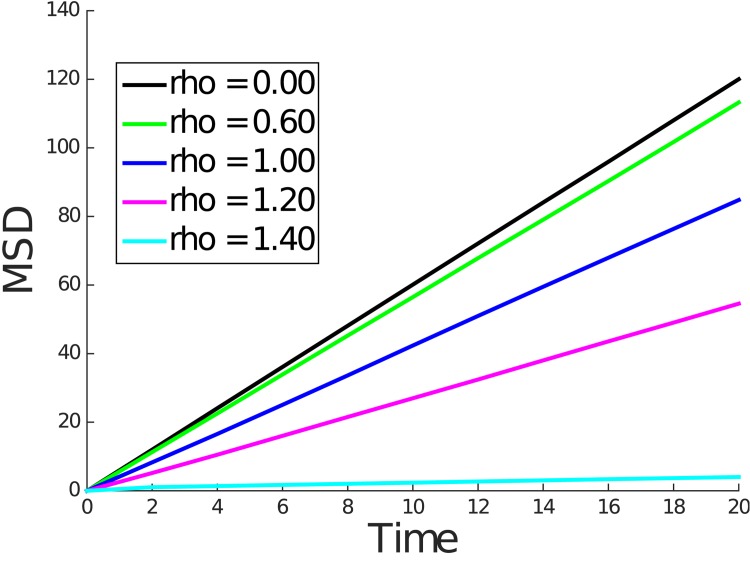
Mean squared displacement for *ρ* = 0.00, 0.60, 1.00, 1.20, and 1.40 for kinetic model simulation.

**Fig 5:**
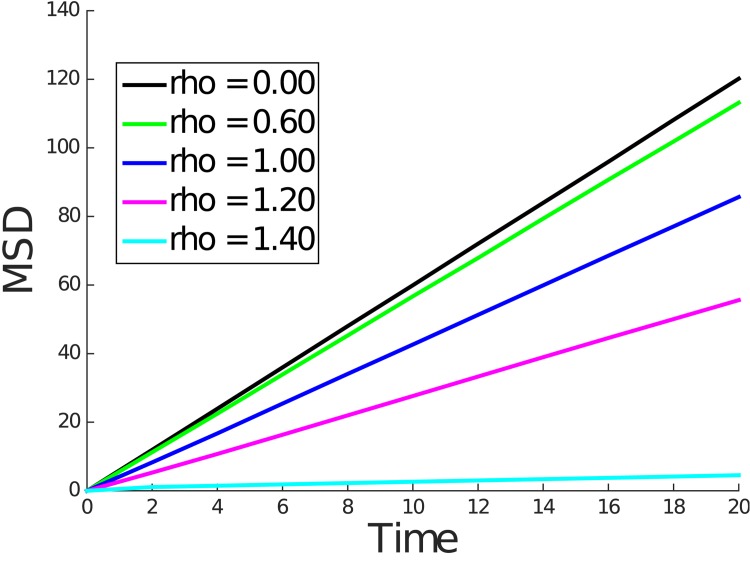
Mean squared displacement for *ρ* = 0.00, 0.60, 1.00, 1.20, and 1.40 for Wiener process simulation.

Figs 4 and 5 contain plots of the mean squared displacement from the kinetic and Wiener process simulations for representative values of *ρ*. These figures show that over longer time periods the mean squared displacement is linear in time, consistent with normal diffusive behavior. We note that the diffusivity which is proportional to the slope of the line decreases as *ρ* increases.

**Fig 6:**
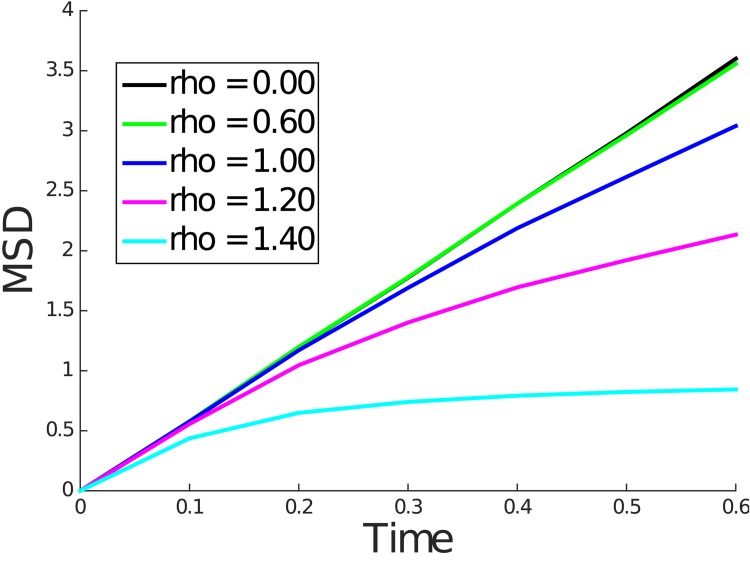
Mean squared displacement for *ρ* = 0.00, 0.60, 1.00, 1.20, and 1.40 for kinetic model simulation until *t* = 0.6.

**Fig 7:**
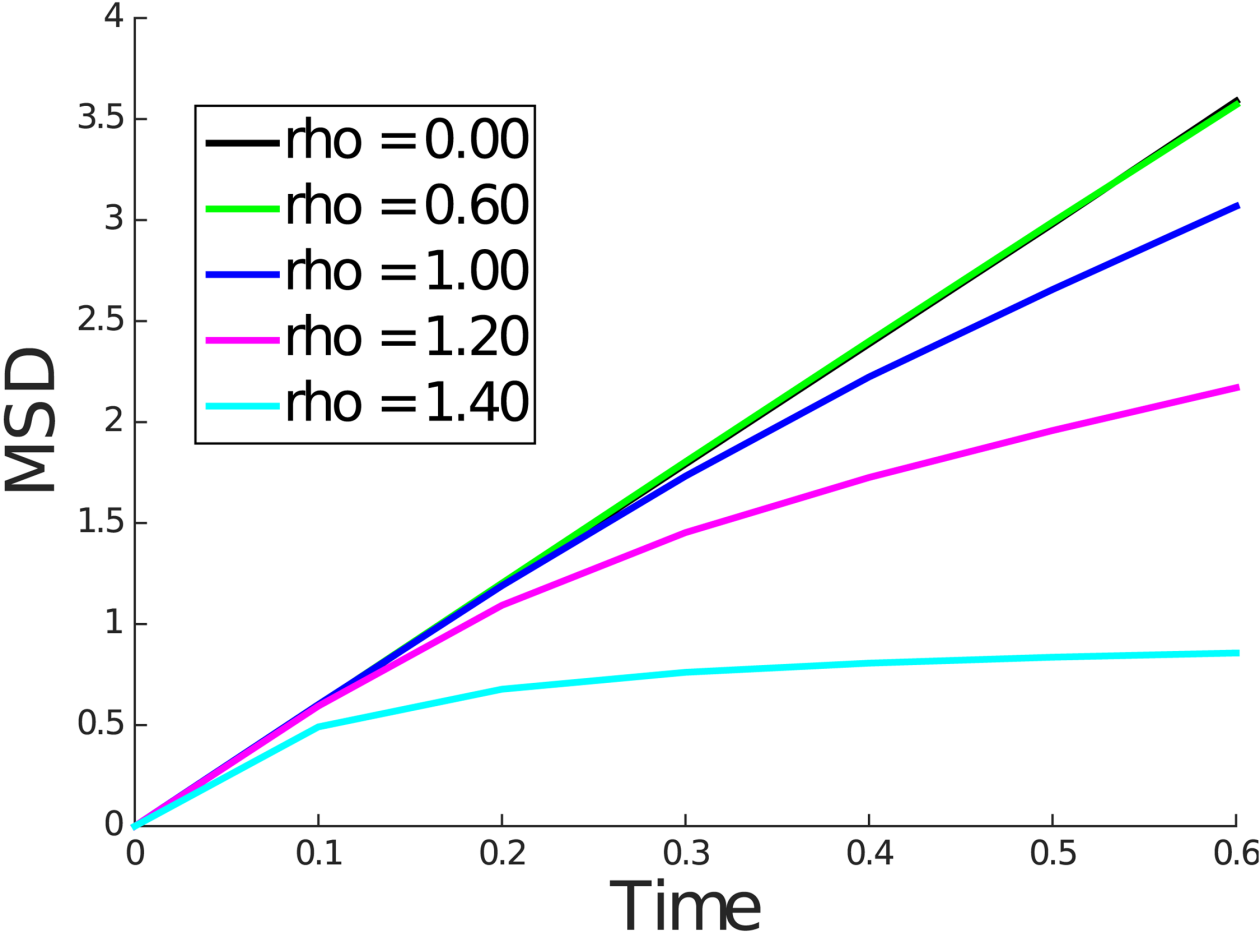
Mean squared displacement for *ρ* = 0.00, 0.60, 1.00, 1.20, and 1.40 for Wiener process model simulation until *t* = 0.6.

For convenience of implementation, in each simulation, the solute molecule started equidistant from the nearest polymer molecules, which caused the solute to diffuse freely until the first collision with a polymer molecule. Thus, we expect the mean squared displacement curve to coincide with the case of the free diffusion line near *t* = 0 and eventually transition to a line with smaller slope indicative of hindered diffusion. In fact, this exact behavior can be seen in Figs 6 and 7, which show the mean squared displacement for small *t*. During this transition from free diffusion to hindered diffusion, we observe a nonlinearity.

The above discussion raises the question of whether, over the length scales of the FCS illuminated volume one could expect to see the hindered but normal diffusive behavior predicted by our simulations. In both the kinetic model and Wiener process simulations, hindered but normal diffusive behavior was observed over a length scale roughly equal to twice the obstruction spacing *L* for small values of *ρ*. For larger values of *ρ*, progressively smaller length scales were sufficient to observe this behavior. This can be concluded from computing the root mean square displacement over the simulation interval shown in Figs 6 and 7. For *ρ* values above 0.2 and up to 1.4, we expect *L* to be in the range of 25 to 160 nm (see Table 2). Since the radial dimension *r*_0_ of the FCS illuminated volume is typically 200–300 nm, we can expect to observe the normal but hindered diffusive behavior under FCS. We note that for very dilute solutions where *L* is larger than *r*_0_ the corresponding *ρ* will be very small indicating nearly free diffusion.

**Fig 8:**
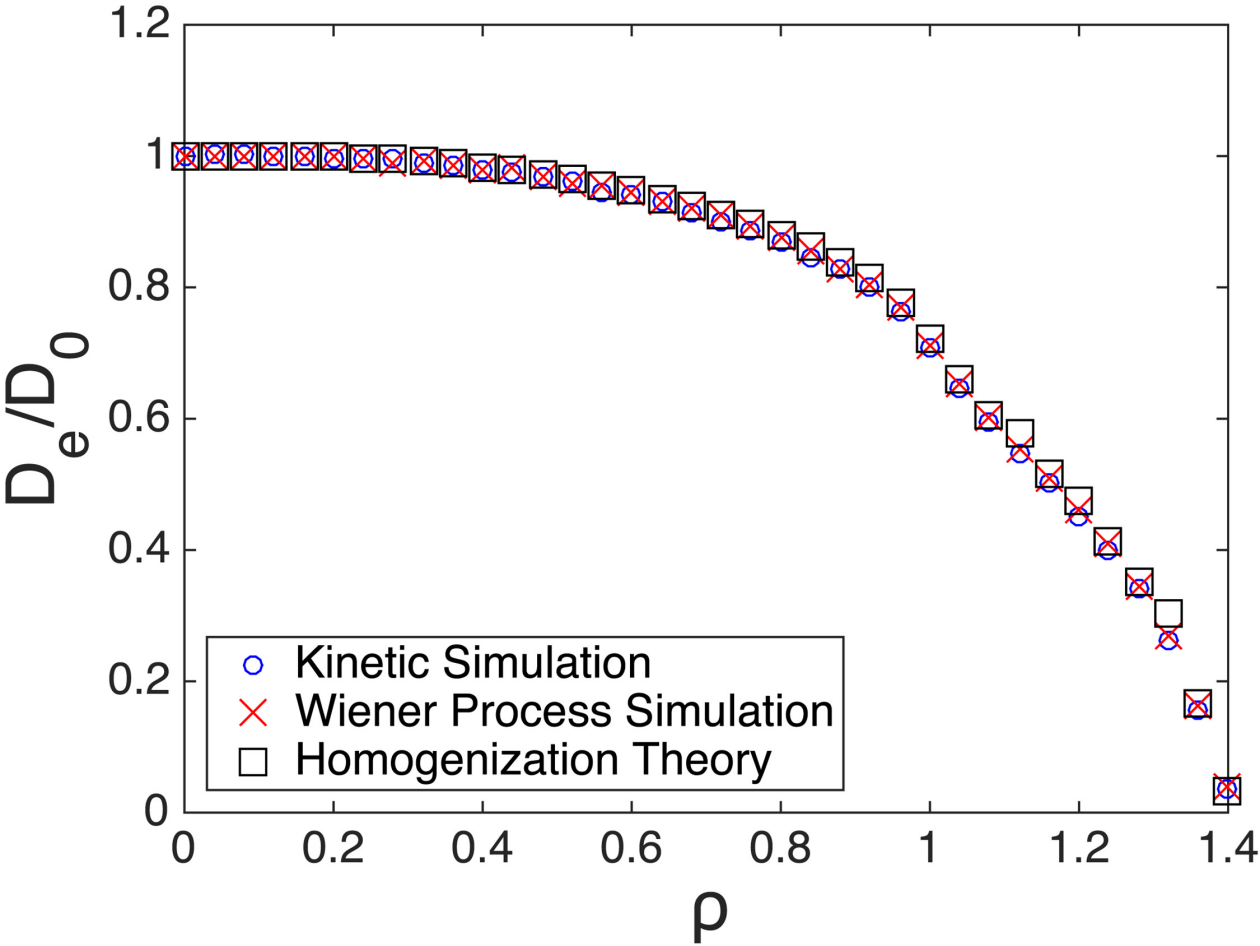
Diffusion coefficient estimates from the Monte Carlo simulations and homogenization theory.

To estimate *D*_*e*_ for a given value of *ρ*, we first computed the line of best fit through the origin of the mean squared displacement and then used $D_{e}=\frac{\text{slope}\;\text{of}\;\text{line}\;\text{of}\;\text{best}\;\text{fit}}{6}$ as the estimate. Fig 8 shows *D*_*e*_ against *ρ* as computed by the Monte Carlo simulations as well as the homogenization computation. S1 Table contains *D*_*e*_ estimates by all three methods and includes the 95% confidence intervals for the Monte Carlo simulations. In all cases, the width of the confidence interval was less than 2% of the estimated value. It is also noteworthy that the *D*_*e*_ estimates of the kinetic and Wiener simulations are within each other’s confidence intervals. Moreover, the value of *D*_*e*_ computed using the homogenization theory is also within the confidence intervals of both Monte Carlo simulations for all *ρ* values computed. Theoretically one expects the homogenization theory to hold in the limit when the ratio *L*/*L*′ approaches zero. In practice, the homogenization result agrees closely with the (more accurate) Wiener process simulations even when *L*′ is only twice the value of *L*. This can be seen from Fig 5 where the square root of the vertical axis provides the length scale *L*′. The straight line approximation over a length scale *L*′ ≈ 2*L* = 10 yielded a value of *D*_*e*_ which agrees closely with the Wiener process simulations. This is a positive result. Additionally, for any given value of *ρ*, 100,000 Wiener process Monte Carlo simulations required around 40 minutes whereas the homogenization theory calculation required less than 1 minute.

#### Anomalous versus hindered but normal diffusion

**Fig 9:**
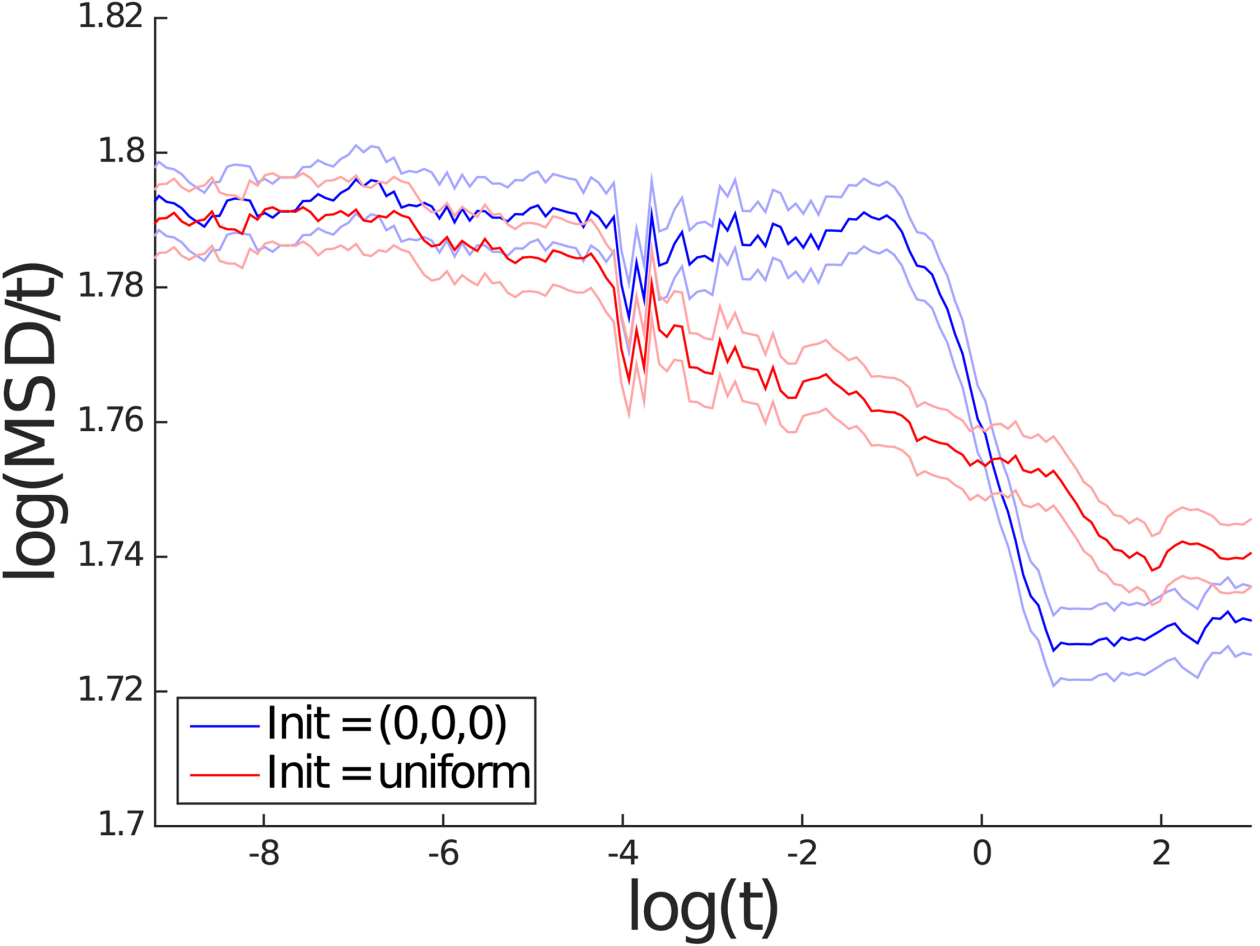
Logarithm of MSD divided by *t* against the logarithm of *t* for *ρ* = 0.60 from the Wiener process simulation. The results in red use an initial position that is uniformly distributed outside the obstacles. The results in blue use an initial position at the origin. The lighter colored lines represent the respective 95% confidence intervals.

**Fig 10:**
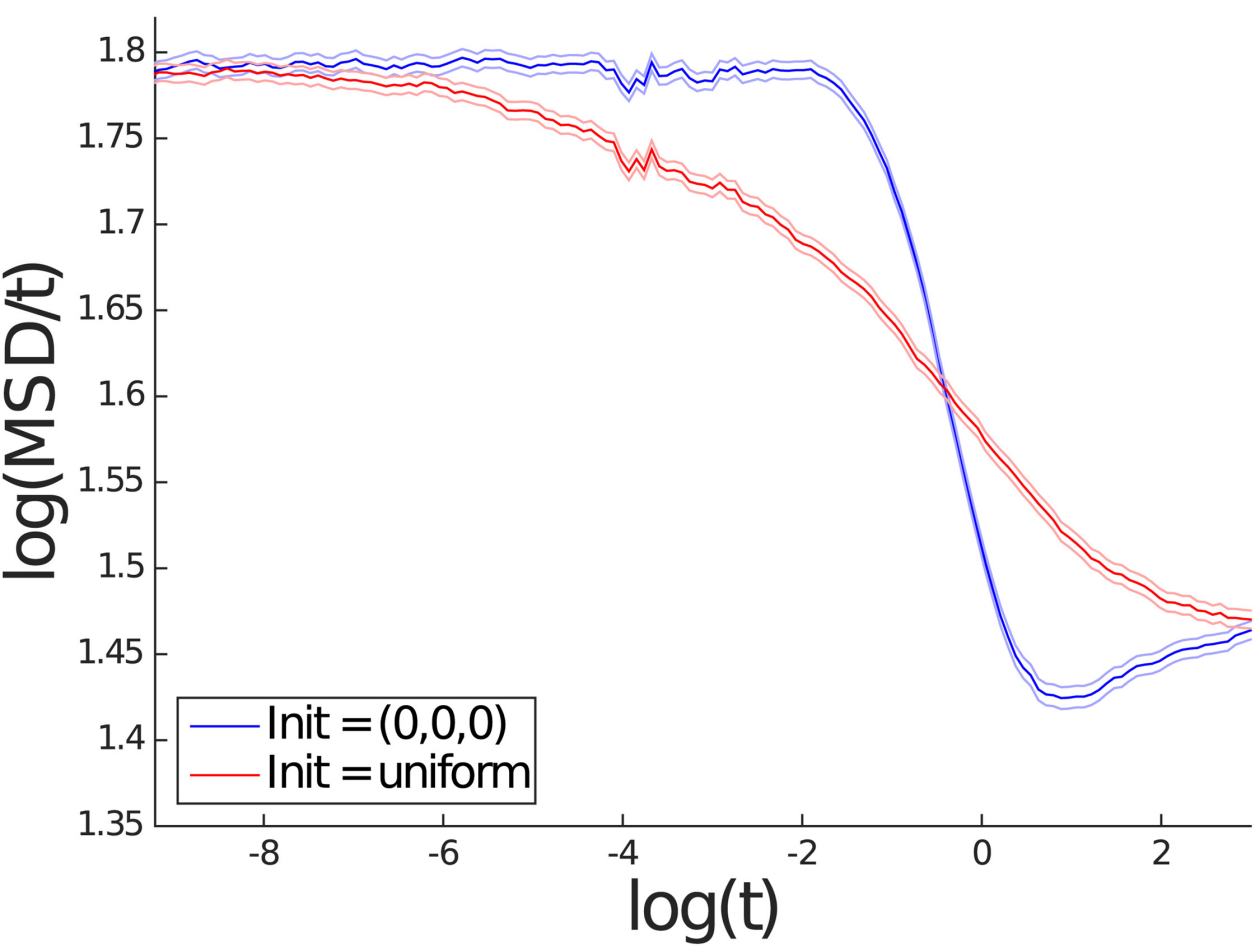
Logarithm of MSD divided by *t* against the logarithm of *t* for *ρ* = 1.00 from the Wiener process simulation. The results in red use an initial position that is uniformly distributed outside the obstacles. The results in blue use an initial position at the origin. The lighter colored lines represent the respective 95% confidence intervals.

It is clear that for the spatial scale of interest to us, the mean square displacement is consistent with normal diffusion. Theoretically, one expects to see free diffusivity *D*_0_ when *t* → 0+ and the effective diffusivity *D*_*e*_ as *t* → ∞, according to Eq (16). In order to take a closer look at how mean squared displacement (MSD) changes over a large range of time scales, for two values of *ρ*, namely *ρ* = 0.6 and *ρ* = 1, MSD was recorded at a wider range of time points. The plots in Figs 9 and 10 show the logarithm of MSD divided by *t* against the logarithm of *t*. Moreover, for each of these two values of *ρ*, two different initial conditions were used: one was as before where the solute starts at a midpoint between obstacles and in the other the solute started out with a random initial condition that was uniformly distributed outside the obstacles. Figs 9 and 10 are in agreement with Eq (16); note that log(6*D*_0_) = log(6)≈1.79. As mentioned earlier, by necessity, the curve log(*MSD*(*t*))/*t* against log(*t*) must transition from one constant value to another. This region of transition does not appear to fit well a power law (i.e. linear in the log scale), and moreover, the transition seems to depend on how the initial condition is chosen.

A transition from anomalous to normal diffusion was observed in [47], though the context there was different (also see [46, 48, 49] for broader studies). The simulations in [47] were carried out in discrete time and as such *t* → 0+ limit does not make much sense. In fact, if a continuous time model was used, one would expect normal diffusive behavior in the *t* → 0+ limit as well. Moreover, the obstruction structure was quite different as the space was discrete and each site was obstructed independently of other sites with a probability of *p*. Bearing in mind that the spacing in the discrete lattice is analogous to the mean free path length of the solute, the spacing between obstructions is comparable to mean free path for, say *p* ≥ 0.2. On the other hand, a lattice based discretization of our model will contain several contiguous lattice sites corresponding to the spherical obstructed regions and several contiguous lattice sites in between these obstructed sites corresponding to the unobstructed region.

Theoretically, one may expect anomalous diffusive behavior as *t* → ∞, i.e. for large time or equivalently spatial scales, when there is no separation of length scales for the spatial structure. In practice, this means at least up to the length scale of observation, the spatial structure has features on every length scale. In the periodic setting that is under consideration here, if the length scale of observation is much larger than the spacing *L*, then one does not expect anomalous diffusion on the large time or spatial scale and our Monte Carlo simulations confirm this. In fact, according to [47], for large *p* values, one observes anomalous diffusion at all time scales. This is because, for *p* values above the percolation threshold, the medium exhibits a fractal structure [47].

The homogenization theory predicts *D*_*e*_/*D*_0_ as a function of *ρ* as depicted in Fig 8. This functional relationship was computed at certain *ρ* values and may be approximated by interpolation for other *ρ* values. We found that the formula

$$\begin{array}{c} D_{e}/D_{0}=e^{-k\rho^{3}} \end{array}$$ (23)

fits this relationship well for smaller *ρ* values. Specifically with *k* = 0.2568 for *ρ* values in the interval [0,0.92] the maximum error between this formula and the curve in Fig 8 was 6.1 × 10^−3^.

**Fig 11:**
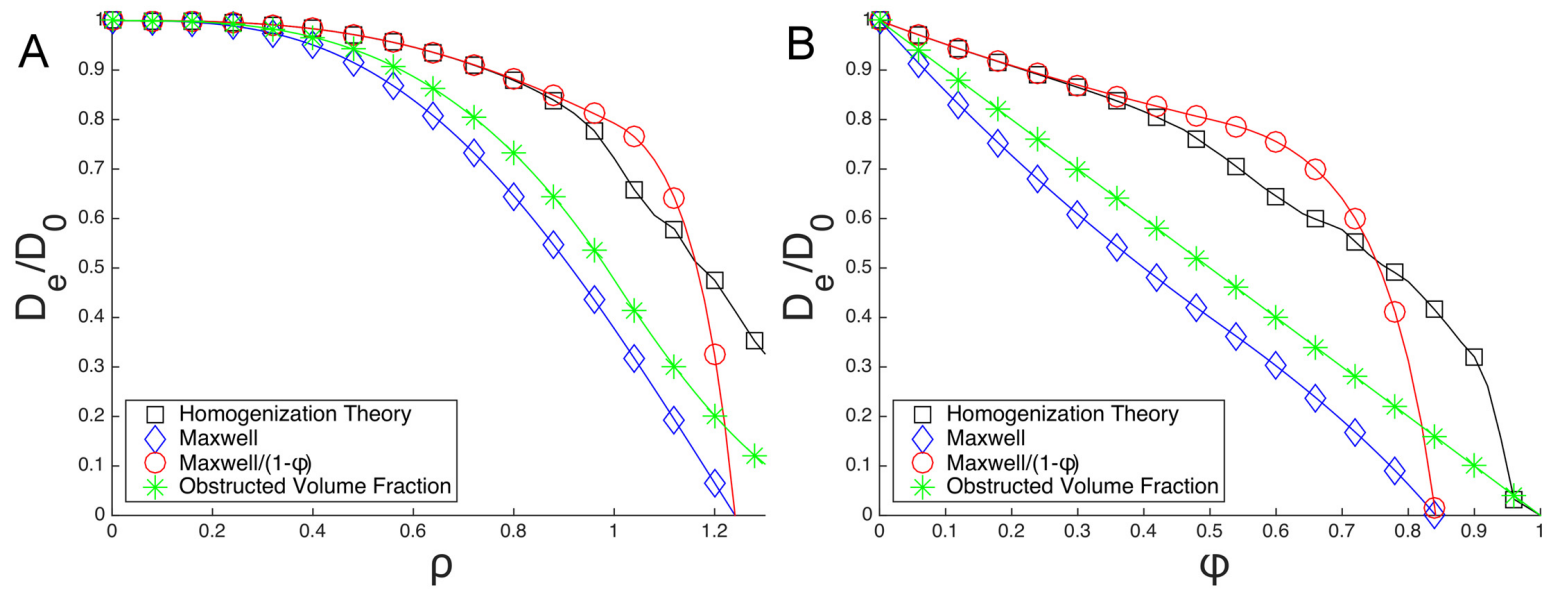
Diffusion coefficients predicted by homogenization theory, Maxwell’s equation, scaled Maxwell’s equation, and the obstructed volume fraction with (a) *ρ* on the x axis, (b) *ϕ* on the x axis.

It is instructive to compare *D*_*e*_/*D*_0_ predicted by homogenization theory with both the naive prediction for *D*_*e*_/*D*_0_ as the straightforward average of diffusivities as well as the Maxwell’s formula given by [28]

$$\begin{array}{c} D_{e}/D_{0}=\frac{1-\frac{L\rho^{3}}{12}}{1+\frac{L\rho^{3}}{24}} \end{array}$$ (24)

where *L* = 4*π*(1 − *δ*)/(2+*δ*) and *δ* is the diffusivity inside the spherical inclusion. For us, *δ* = 0 and hence *L* = 2*π*. Fig 11 shows our homogenization theory prediction in comparison with the naive straightforward average as well as Maxwell’s formula. We note that for *ρ* ≤ 1 the straightforward average is given by the accessible volume fraction 1 − *ϕ* = 1 − *πρ*^3^/6. Additionally, while Maxwell’s formula does not agree with the homogenization theory curve, Maxwell’s formula “corrected” by the factor 1/(1 − *ϕ*) agrees well with the homogenization theory. See the discussion of the factor 1 − *ϕ* in the section on Homogenization Theory.

### Comparison of homogenization results with experimental data

In this work, we used FCS to measure solute diffusivity in three different polymer solutions. The autocorrelation functions for all solute-polymer pairs were described well by a single component fit Eq (20). Even though Eq (20) was originally developed to describe freely diffusing monodisperse fluorescent solutes [34], it has been successfully applied to the description of hindered diffusion in polymer solutions and networks [3, 10, 18]. The assumption of monodispersity was satisfied as each solute molecule was conjugated with a single fluorophore as expected from the 52% labeling efficiency. In all cases, we express the normalized effective diffusivity, *D*_*e*_/*D*_0_, as *τ*_0_/*τ*_*d*_, where *τ*_0_ is the free solute diffusion time in water.

**Fig 12:**
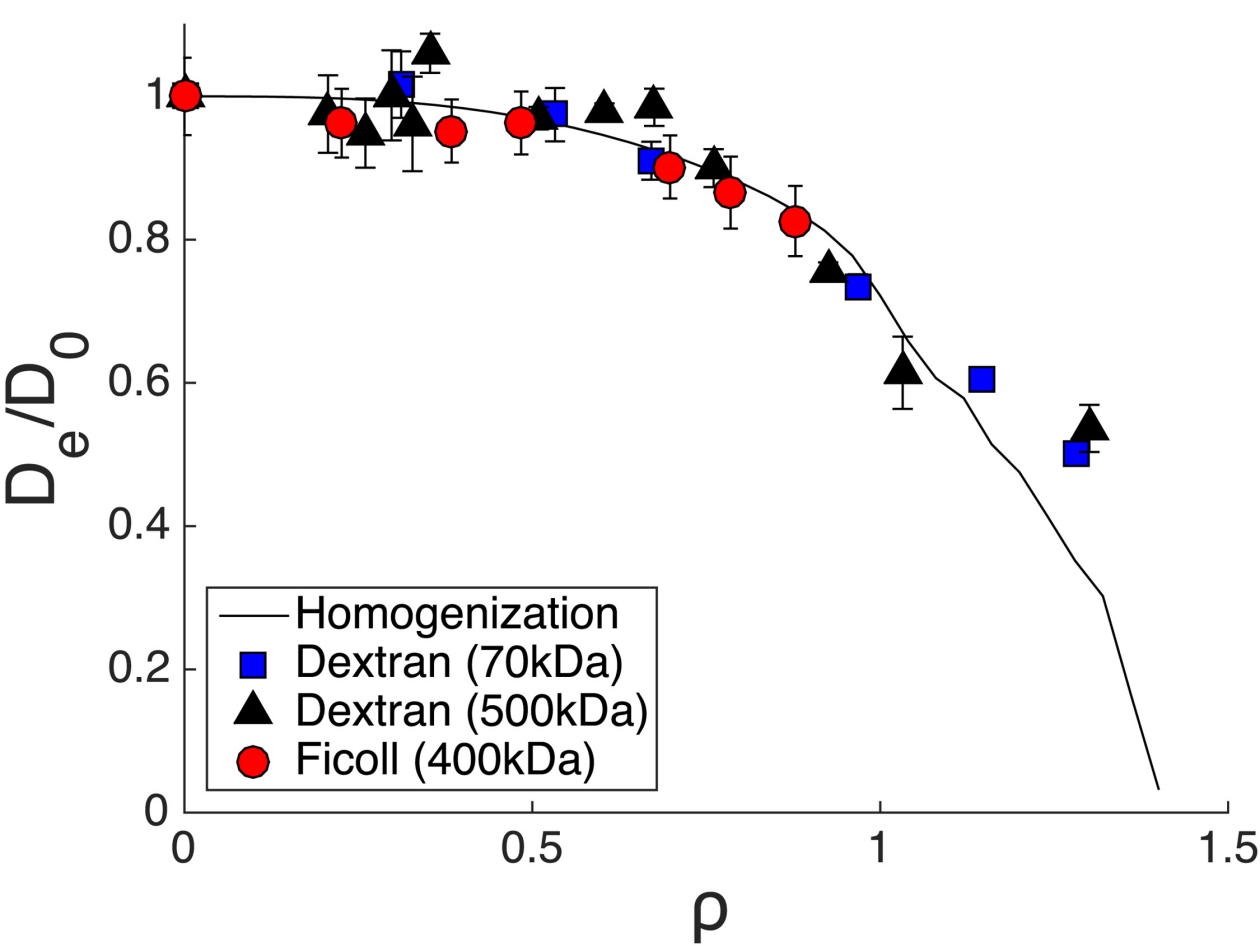
Experimental data from three different polymers is fitted onto the developed homogenization theory model of obstructed diffusion. All polymer solutions were at concentrations up to the overlap concentration, except for data points designated by a circle.

Experimental data on solute diffusivity as a function of polymer concentration for the solute RNase in three different polymer solutions was used to validate the developed model (Fig 12). An increase in polymer concentration leads to a decrease in *L*, which in turn leads to an increase in *ρ* (see Eq (4), Table 2). Note that *ρ* is a lumped dimensionless parameter that is not specific for a given solute-polymer pair but is subject to several assumptions discussed in more detail below. Overall, our data is in excellent agreement with the model indicating that normalized diffusivity decreases with increase in *ρ* due to decrease in spacing between the polymer molecules.

All polymer solutions were prepared in the dilute and semi-dilute regimes, i.e. below and at the overlap concentration Eq (18) unless otherwise noted, to closely match the model assumptions. In particular, the model assumes the polymer obstacles to be rigid spheres meaning that the smallest *L* would be equivalent to the diameter of a single polymer molecule. Based on that geometric model, we define a geometric overlap concentration Eq (19). Note that for polymer molecules such as the dextrans used in this study for which *R*_*g*_ > *R*_*h*_, Eqs (18) and (19) would yield similar overlap concentrations. Due to the rigid sphere assumption, the model is not expected to fit well the diffusivity of solutes in the concentrated regime where chain entanglements are present. To test this assumption, we focus on the diffusivity of RNase in the two dextran solutions, namely Dextran70 and Dextran500. The overlap concentration, c*, is 6.9% w/v (*c*^*θ*^ = 7.4% w/v) and 2.5% w/v (*c*^*θ*^ = 2.5% w/v) for Dextran70 and Dextran500, respectively. We observed an excellent agreement between experimental data and model data for all *ρ* values except for the last data point (circled on the graph) where the decrease in solute diffusivity was less than predicted by the model. The last data point was taken at the concentrated regime for both polymer solutions, specifically a concentration of 7% w/v for Dextran70 and 5% w/v for Dextran500. Thus, as anticipated, we suggest that due to the rigid sphere assumption, the model will predict a faster drop in diffusivity at the concentrated regime expected for a solute diffusing in a solution of flexible polymer chains.

Apart from the rigidity, the second aspect of the assumption is the spherical obstacle geometry. While there are some relatively globular polymers and certainly many globular protein macromolecules, majority of polymers would behave like a random flexible coil. Thus, to test if the model would be applicable to both globular and random coil macromolecules, we chose two types of polymers, namely Ficoll and Dextran (Fig 12). Ficoll is a rigid, highly-branched globular polymer of epichlorohydrin and sucrose, which has been traditionally assumed nearly perfectly spherical in shape of [50] and only more recently it has been described as an intermediate between a solid sphere and a well-solvated linear random coil [51]. Dextran, a slightly branched polymer of 1,6-glucopyranose units, is less compact than Ficoll and behaves like a flexible coil rather than a sphere in an aqueous solution [52]. Interestingly, in all cases, our data matches the model closely: the goodness of the fit was also confirmed by the high values for the coefficient of determination *R*^2^ (Table 3). Note that the *R*^2^ values do not include the two data points in the concentrated regime. While the results indicate that the model could potentially apply to random coil polymers, the homogenization theory can also be modified to describe random coil geometry.

**Table 3:** Coefficient of determination to establish the goodness of the fit between experimental data and the homogenization model data. Note that data in the concentrated regime was excluded from the calculation.

Polymer	*R*^2^
Dextran70	0.95
Ficoll400	0.88
Dextran500	0.89

The model also assumes the obstacles to be stationary, which is a good approximation if the polymer molecule is much larger than the solute and, thus, its diffusivity can be considered negligible as compared to the diffusivity of the solute. Free diffusivity of solutes as a function of solute properties depends on the radius of the solute and its molecular weight [11]. Table 1 depicts the hydrodynamic radii of both the polymer and solute molecules demonstrating that the difference between the two ranges from ∼3-fold (for the RNase-Dextran70 solute-polymer pair) to ∼9-fold (for the RNase-Dextran500 solute-polymer pair). Molecular weights between solute and polymer pairs are also disparate. Thus, the chosen solute-polymer pairs satisfy this assumption.

Lastly, the model assumes no interaction between the solute and polymer: solute diffusivity in the polymer solution is hindered by obstruction only. Thus, solute-polymer pairs were chosen accordingly. For example, we have previously shown that diffusivity of RNase in neutral dextran solution (the one used in this study) is hindered by obstruction only [3]. Ficoll, similarly to dextran, is also an inert sugar-based polymer and interaction between Ficoll and the solute was not expected [53].

## Conclusions

We described a framework based on homogenization theory for the prediction of macro-scale diffusivity of a solute in aqueous polymer solutions. The framework consists of starting with a fine-scale model with clearly stated assumptions and then applying homogenization theory to compute the effective macro-scale diffusivity. Our fine-scale model assumed the polymer molecules to be impenetrable stationary spherical obstacles that were periodically placed in water. The solute was assumed to be a rigid sphere undergoing a Wiener process with specular reflections at the spherical obstacles.

The success of this approach depended on both the accuracy of the fine-scale model as well as the validity of the homogenization theory over the length-scale of interest. Since homogenization theory is only accurate in the limit when the ratio *L*/*L*′ approaches zero, where *L* is the characteristic fine-scale length and *L*′ is the characteristic macro-scale length, the accuracy of the homogenization theory was tested against Monte Carlo simulations of the reflected Wiener process model. We note that for purposes of comparison with FCS experiments, *L*′ is the radial dimension of the FCS confocal volume. However, in applications of longterm interest to us, *L*′ is much larger than this.

Additionally, as a verification of the reflected Wiener process model assumption, we performed a kinetic Monte Carlo simulation. Here we used physically relevant mean path-lengths and did not assume a Gaussian distribution. Both Monte Carlo simulations agreed with each other as well as with the homogenization theory prediction over spatial scales of interest to us. It is also important to note that the homogenization computations are significantly more efficient to carry out than Monte Carlo simulations. Agreement between the two different Monte Carlo simulations with homogenization theory does not validate the fine-scale model, since all three of these computations assumed stationary impenetrable spherical geometry for the polymer molecules. Thus, the most important result we observed was the agreement of the homogenization theory with experimental data for effective solute diffusivities in dilute and semi-dilute regimes.

We note that the homogenization theory predictions also agree with a modified form of the simple Maxwell’s formula for reasonable range of obstruction radii. While the spherical obstruction assumption is simple, and hence the modified Maxwell’s formula could also be used, the main goal of this work is to demonstrate, as a proof-of-principle, the application of the homogenization theoretic framework to the problem of predicting effective diffusivities of a solute in an aqueous polymer molecular environment. To that end, the success of this work shows promise for future work of modeling solute movement in polymer gels using a similar framework. Since the geometry of the gels will be more complicated, simple formulae such as Maxwell’s will not be applicable in those situations and the power of the homogenization theory will be relevant.

## Supporting Information

S1 Text Description of the Monte Carlo simulation algorithm for the kinetic and Wiener process models. (PDF)

S2 Text Description of the confidence interval calculations for the slope of the mean squared displacement. (PDF)

S1 Table Effective diffusivity coefficients from the Monte Carlo simulations and homogenization theory. Diffusion coefficient estimates from the Monte Carlo simulations and COMSOL Multiphysics. In COMSOL Multiphysics, the “fine” mesh setting was generally used and there were no warning or error messages. For *ρ* = 1.32, 1.36, and 1.40 the “extra fine” mesh setting was used due to inaccurate results.
(PDF)
