# Supplementary material for: Homogenization Theory for the Prediction of Obstructed Solute Diffusivity in Macromolecular Solutions
Source: PLoS One. 2016 Jan 5;11(1):e0146093. doi: 10.1371/journal.pone.0146093 (PMC4701423; doi:10.1371/journal.pone.0146093)
Supplement: S1 Text — (PDF) [file pone.0146093.s001.pdf]

S1 Text

Description of the Monte Carlo simulation algorithm for the kinetic and Wiener process models.

Set $t_0 = 0$, $X(t_0) = (0, 0, 0)$, $i = 0$.

While $t_i \leq T$:

If kinetic simulation, choose:

Path length: $l \sim \text{Exponential}(\lambda)$

Time step: $\Delta t \sim \text{Exponential}(\tau)$

Direction: dx, dy, dz uniform r.v.

Position change: $\Delta X = (dx, dy, dz)$ and normalize so that $||\Delta X|| = l$

If Wiener process simulation, choose:

Time step:

$$\Delta t = \left(\frac{L}{2D_0}\right)^2 \times 10^{-4} \quad \text{fixed constant}$$

Direction: $dx, dy, dz \sim \mathcal{N}(0, \Delta t)$

Position change: $\Delta X = (dx, dy, dz)$

$t_{i+1} = t_i + \Delta t$

$X(t_{i+1}) = X(t_i) + \Delta X$

If the path from $X(t_i)$ to $X(t_{i+1})$ intersects an obstruction:

compute reflected path (specular reflection) to determine correct $X(t_{i+1})$

$i = i + 1$

end
