# Supplementary material for: Homogenization Theory for the Prediction of Obstructed Solute Diffusivity in Macromolecular Solutions
Source: PLoS One. 2016 Jan 5;11(1):e0146093. doi: 10.1371/journal.pone.0146093 (PMC4701423; doi:10.1371/journal.pone.0146093)
Supplement: S2 Text — (PDF) [file pone.0146093.s002.pdf]

S2 Text

Description of the confidence interval calculations for the slope of the mean squared displacement.

Let $Y_{il} = ||X_l(t_i)||^2$ denote the squared displacement of the lth trajectory at the ith time point for $l = 1, ..., N$ and $i = 1, ..., m$. The sample mean squared displacement $\bar{Y}_i$ at the ith time point is computed by

$$\bar{Y}_i = \frac{1}{N}\sum_{l=1}^{N} Y_{il}, \tag{1}$$

which provides an estimate of the expected value $E(Y_{il})$. We also denote by C_{ij} the sample covariance between the square displacements at times t_i and t_j, where C_{ij} are given by

$$C_{ij} = \frac{1}{N}\sum_{l=1}^{N}(Y_{il} - \bar{Y}_i)(Y_{jl} - \bar{Y}_j) = \left(\frac{1}{N}\sum_{l=1}^{N} Y_{il}Y_{jl}\right) - \bar{Y}_i\bar{Y}_j, \tag{2}$$

and provide estimates of $\mathrm{Cov}(Y_{il}, Y_{jl})$ the covariance between Y_{il} and Y_{jl}.

The slope s of the least squares line through the origin is given by

$$s = \sum_{i=1}^{m} \alpha_i \bar{Y}_i, \tag{3}$$

where

$$\alpha_i = \frac{t_i}{\sum_{j=1}^{m} t_j^2}. \tag{4}$$

In order to calculate the 95% confidence intervals for the estimated slope s we use the formula for the variance $\mathrm{Var}(s)$ of s given by

$$\mathrm{Var}(s) = \sum_{i=1}^{N}\sum_{j=1}^{N} \alpha_i\alpha_j \mathrm{Cov}(\bar{Y}_i, \bar{Y}_j), \tag{5}$$

where $\mathrm{Cov}(\bar{Y}_i, \bar{Y}_j)$ is the covariance between $\bar{Y}_i$ and $\bar{Y}_j$. Since

$$\mathrm{Cov}(\bar{Y}_i, \bar{Y}_j) = \frac{1}{N}\mathrm{Cov}(Y_{il}, Y_{jl}), \tag{6}$$

we estimate $\mathrm{Cov}(\bar{Y}_i, \bar{Y}_j)$ by C_{ij}/N. This gives the formula

$$V = \frac{1}{N}\sum_{i=1}^{N}\sum_{j=1}^{N} \alpha_i\alpha_j C_{ij}, \tag{7}$$

for the estimated variance V of s. Finally, the 95% confidence interval for the slope is computed by

$$CI = (s - 1.96\sqrt{V}, s + 1.96\sqrt{V}). \tag{8}$$
