# Supplementary material for: Homogenization Theory for the Prediction of Obstructed Solute Diffusivity in Macromolecular Solutions
Source: PLoS One. 2016 Jan 5;11(1):e0146093. doi: 10.1371/journal.pone.0146093 (PMC4701423; doi:10.1371/journal.pone.0146093)
Supplement: S1 Table — Diffusion coefficient estimates from the Monte Carlo simulations and COMSOL Multiphysics. In COMSOL Multiphysics, the “fine” mesh setting was generally used and there were no warning or error messages. For ρ = 1.32, 1.36, and 1.40 the “extra fine” mesh setting was used due to inaccurate results. (PDF) [file pone.0146093.s003.pdf]

S1 Table

Effective diffusivity coefficients from the Monte Carlo simulations and homogenization theory.

ρ	D_e (Kinetic)	D_e (Wiener)	D_e (Homog.)
0.00	1.000566±0.004387	1.000861±0.004440	1.000000
0.04	0.997985±0.004381	1.002199±0.004408	0.999984
0.08	1.000120±0.004407	1.001246±0.004404	0.999867
0.12	1.000591±0.004403	1.000679±0.004413	0.999551
0.16	0.999836±0.004386	0.998861±0.004408	0.998936
0.20	0.998996±0.004391	0.997686±0.004376	0.997923
0.24	0.995379±0.004356	0.995253±0.004386	0.996410
0.28	0.989591±0.004323	0.995455±0.004368	0.994307
0.32	0.991516±0.004367	0.989250±0.004341	0.991521
0.36	0.986247±0.004315	0.985974±0.004347	0.987958
0.40	0.979761±0.004294	0.980290±0.004342	0.983547
0.44	0.980653±0.004314	0.976118±0.004296	0.978212
0.48	0.968987±0.004273	0.969549±0.004290	0.971891
0.52	0.959893±0.004227	0.960342±0.004238	0.964522
0.56	0.953480±0.004184	0.945978±0.004203	0.956056
0.60	0.943733±0.004181	0.941921±0.004156	0.946461
0.64	0.931924±0.004117	0.930894±0.004145	0.935683
0.68	0.922267±0.004100	0.912034±0.004030	0.923685
0.72	0.908800±0.004034	0.901135±0.004012	0.910389
0.76	0.891593±0.003947	0.886619±0.003971	0.895656
0.80	0.876595±0.003924	0.868575±0.003867	0.879243
0.84	0.854856±0.003844	0.845554±0.003781	0.860673
0.88	0.829640±0.003725	0.828839±0.003750	0.839058
0.92	0.804680±0.003654	0.798938±0.003642	0.812635
0.96	0.771317±0.003546	0.761917±0.003505	0.777616
1.00	0.712882±0.003310	0.706465±0.003311	0.721637
1.04	0.654620±0.003096	0.645945±0.003087	0.658307
1.08	0.601318±0.002888	0.595037±0.002887	0.606641
1.12	0.555165±0.002731	0.545697±0.002676	0.578675
1.16	0.507953±0.002534	0.502729±0.002534	0.514373
1.20	0.462473±0.002390	0.452299±0.002338	0.475340
1.24	0.408230±0.002199	0.399985±0.002167	0.414279
1.28	0.344835±0.001963	0.340391±0.001970	0.351803
1.32	0.268164±0.001693	0.261548±0.001665	0.302295
1.36	0.163680±0.001259	0.157692±0.001236	0.165528
1.40	0.040768±0.000545	0.036536±0.000503	0.033331

Table 1: Diffusion coefficient estimates from the Monte Carlo simulations and COMSOL Multiphysics. In COMSOL Multiphysics, the "fine" mesh setting was generally used and there were no warning or error messages. For $\rho = 1.32$, 1.36, and 1.40 the "extra fine" mesh setting was used due to inaccurate results.
